# 非小细胞肺癌患者尿蛋白质组差异表达分析

**DOI:** 10.3779/j.issn.1009-3419.2015.03.03

**Published:** 2015-03-20

**Authors:** 振岗 陈, 金波 刘, 凌 林, 辉 谢, 文成 张, 洪博 张, 广舜 王

**Affiliations:** 301800 天津，天津医科大学宝坻临床学院肿瘤外科 Department of Oncology Srugery, Baodi Clinical Hospital, Tianjin Medical University, Tianjin 301800, China

**Keywords:** 肺肿瘤, 蛋白质组学, 尿液, 生物标记物, Lung neoplasms, Proteomics, Urine, Biomarker

## Abstract

**背景与目的:**

筛查非小细胞肺癌（non-small cell lung cancer, NSCLC）患者尿液中差异表达蛋白，确定可用于NSCLC早期诊断、监测预后和治疗评估的生物标记物。

**方法:**

分别收集40例已病理证实初诊NSCLC患者、8例肺部良性疾病患者和22例健康志愿者的尿液样本。利用0.9%一维十二烷基硫酸钠-聚丙烯酰胺凝胶电泳（sodium dode-cyl sulfate polyacrylamide gel electrophoresis, 1D SDS-PAGE）技术和MS-Thermo-Orbitrap-Velos质谱分析仪对NSCLC组和非肿瘤组尿液中蛋白质进行分离、提取及识别，鉴定出NSCLC患者尿液中的差异表达蛋白。应用SPSS 20.0软件中受试者工作特征曲线（receiver operating characteristic curve, ROC）分别对其敏感性、特异性进行分析，并进行实验验证，从而确定出与NSCLC相关的生物标记物。

**结果:**

NSCLC患者组和非肿瘤组尿液差异性表达蛋白质集中表现在90 kDa、60 kDa和20 kDa-30 kDa凝胶条带中。在NSCLC患者尿液蛋白分析中发现了4种与NSCLC相关的差异表达蛋白，包括上调蛋白LRG1、CA1和下调蛋白VPS4B、YWHAZ。这4种差异表达蛋白作为独立的NSCLC生物标记物其敏感性较低：LRG1蛋白敏感性83.0%（25/30）、特异性90.0%（18/20）；CA1蛋白敏感性60.0%（18/30）、特异性90.0%（18/20)；VPS4B蛋白敏感性73.3%（22/30）、特异性90.0%（18/20）；YWHAZ蛋白敏感性60.0%（18/30）、特异性95.0%（19/20）。而采用蛋白质组合模式对NSCLC进行筛查、诊断，则其敏感性和特异性分别可高达96.7%（29/30）和85%（17/20）。

**结论:**

LRG1、CA1蛋白在NSCLC患者尿液中高表达，而VPS4B、YWHAZ蛋白呈低表达，差异表达蛋白均提示有可能成为用于NSCLC早期筛查、监测预后和治疗评估的生物标记物。LRG1、CA1、VPS4B和YWHAZ尿液蛋白作为单一生物标记物应用于NSCLC筛查和诊断的敏感性较低，而采用蛋白质组合模式明显优于独立模式对NSCLC的筛查和诊断，故蛋白质组合模式在临床诊疗中将更具有良好应用价值和前景。

## Abstract (en)

**Background and objective:**

Screen differentially expressed proteins in patients with non-small cell lung cancer (NSCLC), and aim to identify biomarkers for early screening, monitoring prognosis and evaluating therapy of NSCLC.

**Methods:**

Urinary samples were collected from 40 newly diagnosed NSCLC patients, 8 patients with lung benign disorders and 22 healthy people. 0.9% sodium dodecylsulfate- polyacrylamide gel electrophoresis (1D SDS-PAGE) and MS-Thermo-Orbitrap-Velos were applied to separate, extract and identify proteins in urinary samples from non-neoplastic groups and NSCLC patients, in order to find out differentially expressed proteins in patients with NSCLC. Then, sensitivity and specificity of candidate proteins were tested by certain experiments. Finally, biomarkers related to NSCLC could be determined.

**Results:**

The differences of urinary proteins between non-neoplastic groups and NSCLC patients mainly focused on 90 kDa, 60 kDa and 20 kDa-30 kDa stripes. Four differently expressed proteins were found in urinary proteins in NSCLC group, including LRG1, CA1 (up-regulating proteins) and VPS4B, YWHAZ (down-regulating proteins). The sensitivity of these four proteins for biomarker of NSCLC was relatively low when they were used to screen or diagnose independently. The sensitivity and specificity of LRG1 was 83.0% (25/30) and 90.0% (18/20), respectively; 60.0% (18/30) and 90.0% (18/20) for CA1; 73.3% (22/30) and 90.0% (18/20) for VPS4B; 60.0% (18/30) and 95.0% (19/20) for YWHAZ. However, the sensitivity and specificity would increase to 96.7% (29/30) and 85% (17/20) after the four biomarkers were combined.

**Conclusion:**

LRG1 and CA1 are abundant in urine in patients with NSCLC, while VPS4B and YWHAZ are low-abundance proteins. They could be regarded as biomarkers for early screening, monitoring prognosis and evaluating therapy of patients with NSCLC because of differential expression. The sensitivity of the four biomarkers of NSCLC is relatively low when they are used to screen or diagnose independently, while significantly improvement if they were in combined pattern, which will be of excellent applications to clinical diagnosis and treatment.

肺癌已成为癌症死亡的主要原因^[1]^，且呈逐年上升趋势，而其中非小细胞肺癌（non-small cell lung cancer, NSCLC）约占肺癌总数的80%。虽然不断改善NSCLC的各种诊断和治疗方法，但60%NSCLC患者在接受治疗时已处于晚期^[2]^，其5年生存率不超过15%，而Ⅰ期NSCLC的5年生存率却高达70%^[3]^，故在临床诊治中急需寻找一种NSCLC早期筛查和诊断的新方法。现阶段在NSCLC研究中，一个重要的方向是使用基因和蛋白质组学方法来探讨NSCLC肿瘤细胞的生物学特性。近些年来，通过NSCLC患者尿液中获得差异表达蛋白，从而为NSCLC预防、诊断和治疗提供潜在生物标志物的研究越来越得到科研工作人员的普遍关注。我们通过应用蛋白质组学和生物信息技术对肺部良性疾病、健康志愿者和NSCLC患者尿液中差异表达蛋白质分析，寻找出尿液蛋白LRG1、CA1、VPS4B和YWHAZ为NSCLC早期筛查、监测预后和治疗评估的生物标记物。

## 1 材料和方法

### 1.1 一般资料

来自天津医科大学宝坻临床学院肿瘤科2014年3月-2014年10月病理证实初诊NSCLC患者尿液样本40例。其中男性27例，女性13例；年龄44岁-84岁，平均年龄（64±10.2）岁；腺癌13例，鳞癌27例；Ⅰ期3例，Ⅱ期5例，Ⅲ期20例，Ⅳ期12例。来自天津医科大学宝坻临床学院保健科与肿瘤科对照组尿液样本30例：健康志愿者22例；肺部良性疾病8例，包括肺部错构瘤3例，肺部结核球2例，肺部炎性假瘤1例，肺部阴影2例。其中男性20例，女性10例；年龄24岁-86岁，平均年龄（59±15.5）岁。所有参与者均被告知尿液标本用于研究目的，在医院进行晨起第一次中段尿液收集。受试人主体协议成立，进行内部审查委员会批准。受试者知情同意。

### 1.2 主要试剂及仪器

二硫苏糖醇（DTT）来自北京欣经科生物技术有限公司；尿素来自西陇化学工业有限公司；三氨基甲烷（Tris）、十二烷基硫酸钠（SDS）、丙烯酰胺、过硫酸钠、乙醇均来自北京化学制剂公司；乙腈、碳酸氢铵来自J.D.Baker公司；考马斯亮蓝来自Merck KGaA公司。超高速低温离心机为美国Berkman公司产品；550全自动酶标仪为美国BIO. RAD公司产品，分析软件为Microsoft Excel软件；Protean^TM^ PlusDodeca^TM^ Cell电泳槽、Protean^TM^ PlusDodeca^TM^ Cell染胶仪为美国BIO.RAD公司产品；MS-Thermo-Orbi-Trap-Velos质谱仪为美国热电公司产品。实验软件包括：4000 explorer 3.0软件、GPS explorer 3.5软件和NCBI-Human数据库分析软件。

### 1.3 尿液样本采集处理

分别收取近3年内未发现其他系统严重疾病，近2年无严重疾病用药史，特别是近1个月内无泌尿系统疾病史，女性不在月经周期内肺部良性疾病、志愿者及病理证实初诊NSCLC患者晨起第一次中段尿液样本各40 mL。液氮快速冷冻后，置于-80 ℃冰箱冻藏保存备用。

### 1.4 蛋白质提取及定量测定

溶解冰冻样本时进行多次涡旋，以最大程度的提高蛋白质回收率^[4]^。采用200, 000 g超高速离心沉淀法分离定容为20 mL尿液样本提取其尿液蛋白质，并进行还原、烷基化和裂解。通过Branford法进行蛋白质浓度测定，取出定量为20 μg样本溶液进行蛋白质分离。

### 1.5 一维电泳SDS-PAGE蛋白质分离

取20 μg样本溶液进行电泳分离，了解其差异凝胶条带情况。实验中切去高丰度蛋白质集中的75 kDa凝胶条带，将余下条带等分为4-6等份，进行凝胶内胰蛋白酶消化。最后，分别使用30%乙腈、0.3%三氟乙酸和100%乙腈进行胰蛋白酶肽段的萃取。

### 1.6 差异表达蛋白质识别

使用MS-Thermo-Orbi-Trap-Velos质谱分析仪对蛋白质及肽段逐一予以分离并进行鉴定，所得质谱数据通过进入HCBI-Human数据库进行数据分析与蛋白质识别，得出全部尿液蛋白质。同时对所得蛋白质数据进行搜索和分析，分别筛选出在NSCLC患者尿液中的上调和下调蛋白质。最后，应用SPSS 20.0软件中受试者工作特征曲线（receiver operating characteristic curve, ROC）对筛选出蛋白质的敏感性和特异性进行统计学分析，确定出与NSCLC相关的差异表达蛋白质，初步认定为NSCLC早期筛查的候选生物学标记物。

### 1.7 差异蛋白敏感性、特异性验证

双盲法对20例验证实验样本进行同上处理，对所得结果进行统计分析，得出差异表达蛋白对NSCLC早期诊断的敏感性和特异性。

### 1.8 统计学方法

采用SPSS 20.0统计软件进行数据统计与分析。

## 2 结果

### 2.1 1DSDS-PAGE电泳凝胶图结果

非肿瘤组和NSCLC组尿液蛋白的差异表达主要出现在90 kDa、60 kDa和20 kDa-30 kDa凝胶条带，而高丰度蛋白质集中于75 kDa条带处（图 1）。

**1:**
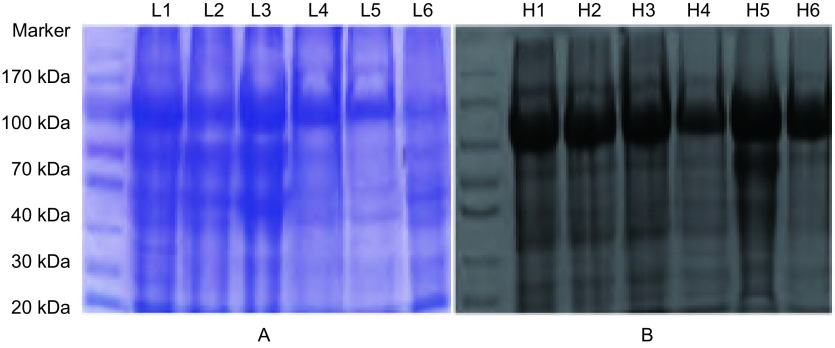
NSCLC组与非肿瘤组尿液蛋白质的电泳图像。A：6例NSCLC患者尿液蛋白电泳图；B：6例非肿瘤组尿液蛋白电泳图。 The electrophoresis images of urine proteins from NSCLC patients and non-neoplastic groups. A: The electrophoresis images of urine proteins from 6 NSCLC patients; B: The electrophoresis images of urine proteins from 6 non-neoplastic patients.

### 2.2 质谱数据采集结果

通过MS-Thermo-Orbitrap-Velos质谱仪进行尿液蛋白质提取分离，所得蛋白质和肽段结果如下：实验中NSCLC组鉴别出肽段数6, 789-9, 645，中位数7, 762（7, 189-8, 672）；蛋白质数1, 194-2, 813，平均值2, 001±363.1。非肿瘤组鉴别出肽段数6, 277-8, 700，中位数7, 774（6, 452-8, 616）；蛋白质数1, 313-2, 774，平均值1, 967±505.0。双盲实验中NSCLC组鉴别出肽段数5, 919-9, 045，平均值7, 705±1, 112.6；蛋白质数1, 194-2, 408，平均数1, 937±391.5。非肿瘤组鉴别出肽段数6, 132-9, 376，平均值7, 870±1, 050.2；蛋白质数1, 060-2, 397，平均数1, 876±409.9。实验与验证实验各组所提取分离出的总平均肽段数为7, 495±1, 091.4，蛋白质总数1, 874±416.8，均满足本实验研究的要求，各组肽段与蛋白质数量分布见图 2。

**2:**
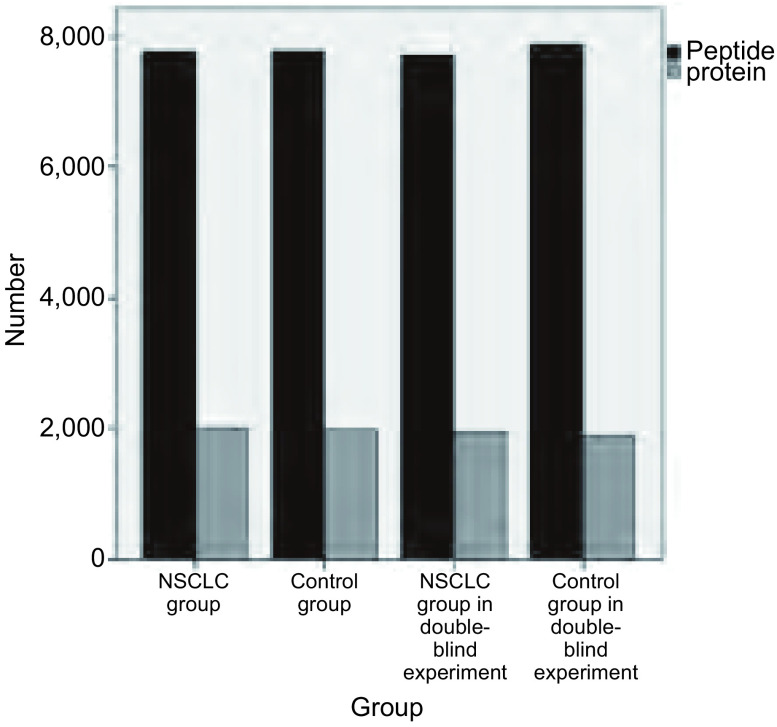
实验中各组样本提取分离出的尿液蛋白质和肽段数 The protein and peptideextracted from urine samples in different groups

### 2.3 MS-Thermo-Orbitrap-Velos质谱仪结果分析

得到的MS/MS数据进行NCBI-Human数据库搜索，提取、确定出尿液样本中包含的所有蛋白质。实验中通过align数据分析软件设定蛋白质肽段数≥2个肽段、蛋白质上调比率＞4倍条件，于NSCLC患者尿液蛋白质中筛查出LRG1（25/30）、CA1（18/30）、PTGDS（23/30）、CD14（20/30）、ERLIN2（21/30）、ACTG1（21/30）6种明显上调蛋白质。同样，设定蛋白质肽段数≥2个肽段、蛋白质下调比率＞4倍条件，于所有NSCLC患者尿液蛋白质中筛查出VPS4B（22/30）、IST1（23/30）、GNAI1（23/30）、UBB（20/30）、YWHAZ（18/30）5种明显下调蛋白质。最后，应用SPSS 20.0软件分别对上调蛋白质、下调蛋白质的敏感度和特异度进行ROC曲线图分析，发现上调蛋白LRG1、CA1（图 3，表 1）、下调蛋白VPS4B、YWHAZ（图 4，表 2）的敏感性和特异性较高，曲线下面积均大于0.75，具有统计学意义。因此，考虑此4种尿液差异表达蛋白与NSCLC具有相关性，初步将其确定为NSCLC早期筛查的候选生物学标记物。

**3:**
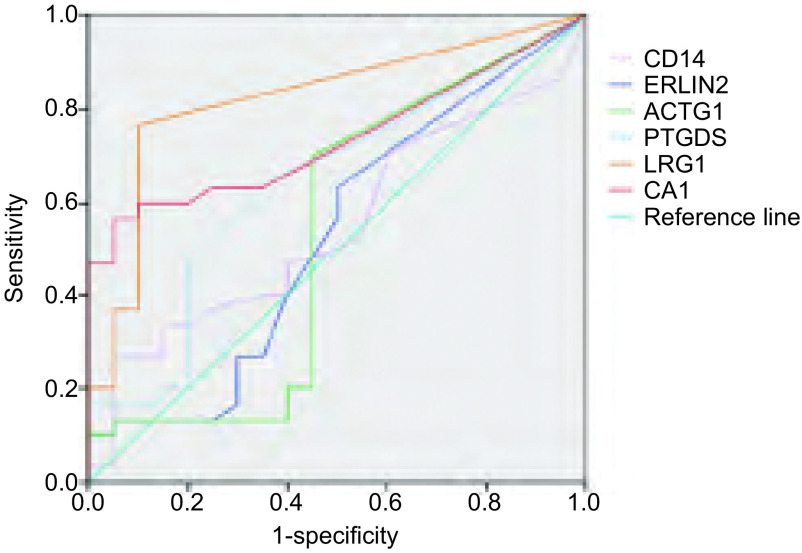
上调蛋白质ROC分析曲线图 ROC curve of up-regulating proteins

**1:** 上调蛋白质ROC曲线下面积 Area under ROC curve of up-regulating proteins

Types	Area	SEM^a^	*P*^b^	95%CI
LRG1	0.823	0.063	< 0.001	0.701-0.946
CA1	0.737	0.070	0.005	0.600-0.874
PTGDS	0.644	0.080	0.087	0.487-0.802
CD14	0.548	0.082	0.566	0.387-0.709
ACTG1	0.529	0.092	0.729	0.349-0.710
ERLIN2	0.526	0.087	0.759	0.356-0.695
^a^: under non-parametric hypothesis; ^b^: null hypothesis: actual area=0.5.				

**4:**
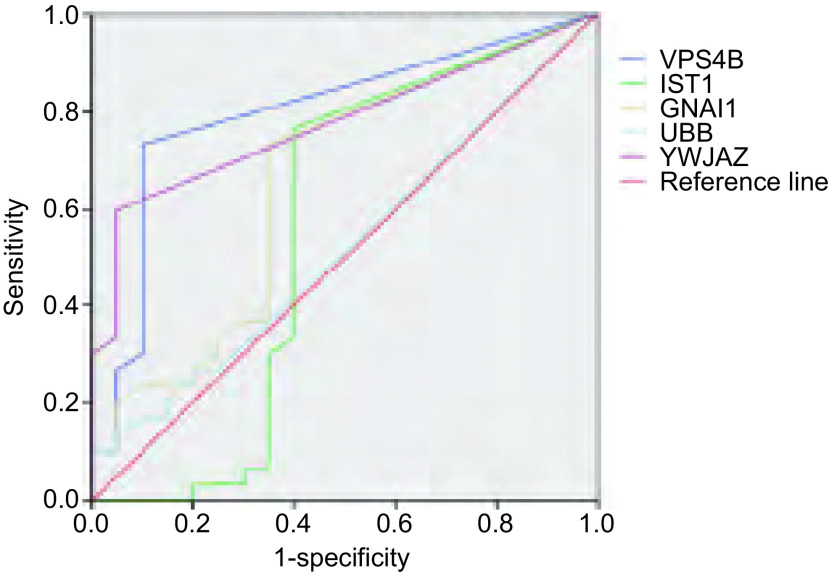
下调蛋白质R0C分析曲线图 ROC curve of down-regulating proteins

**2:** 下调蛋白质ROC曲线下面积 Area under ROC curve of down-regulating proteins

Types	Area	SEM^a^	*P*^b^	95%CI
VPS4B	0.799	0.067	< 0.001	0.667-0.931
YWHAZ	0.776	0.066	0.001	0.647-0.905
GNAI1	0.653	0.082	0.070	0.491-0.814
IST1	0.553	0.098	0.533	0.361-0.744
UBB	0.521	0.083	0.804	0.358-0.684
^a^: under non-parametric hypothesis; ^b^: null hypothesis: actual area=0.5.				

### 2.4 差异表达蛋白的敏感性、特异性

LRG1和CA1蛋白在NSCLC患者尿液中呈现高表达，LRG1蛋白敏感性83.0%（25/30）、特异性90.0%（18/20）；CA1蛋白敏感性60.0%（18/30）、特异性90.0%（18/20）。VPS4B和YWHAZ蛋白在NSCLC患者尿液中呈现低表达，VPS4B蛋白敏感性73.3%（22/30）、特异性90.0%（18/20）；YWHAZ蛋白敏感性60.0%（18/30）、特异性95.0%（19/20）；LRG1、CA1、VPS4B和YWHAZ蛋白质组合模式的敏感性和特异性分别为96.7%（29/30）和85%（17/20），见表 3，表 4。在本研究中蛋白质组敏感性定义为各个生物标记物所提示的NSCLC患者均纳入总数，特异性定义为不出现任何一种生物标记物的实验对象均纳入总数。

**3:** 独立差异蛋白质敏感性和特异性 Sensitivity and specificity of differentially expressed proteins

Proteins	NSCLC group	Control group	Sensitivity (%)	Specificity (%)	*P*
LRG1	25/30	18/20	83.0	90.0	0.003
CA1	18/30	18/20	60.0	90.0	0.003
VPS4B	22/30	18/20	73.3	90.0	0.007
YWHAZ	18/30	19/20	60.0	95.0	< 0.001

**4:** 差异表达蛋白质组敏感性和特异性 Sensitivity and specificity of differentially expressed proteome

Group	NSCLC	Non-neoplastic	Sensitivity (%)	Specificity (%)	*P*
NSCLC (*n*=30)	29	1	96.7	85.0	0.017
Non-neoplastic (*n*=20)	3	17			

### 2.5 验证实验结果

10例NSCLC样本中正确筛查出了9例NSCLC患者；10例非肿瘤样本中正确排除7例。采用差异表达蛋白质组合模式双盲下对NSCLC患者进行筛查和诊断，其敏感性和特异性分别为90.0%（9/10）和70.0%（7/10），其预测的阳性预测值（positive predictive value, PPV）和阴性预测值（negative predictive value, NPV）分别高达75.0%和87.5%（表 5）。

**5:** 验证实验的敏感性和特异性 Sensitivity and specificity in double-blind experiment

Group	NSCLC	Non-neoplastic	Sensitivity (%)	Specificity (%)	*P*
NSCLC (*n*=10)	9	1	90.0	70.0	0.041
Non-neoplastic (*n*=10)	3	7			

## 3 讨论

在男性和女性中肺癌已成为全世界癌症死亡的主要原因，每年有超过100万人死于肺癌^[5]^。NSCLC约占肺癌患者中的80%，在过去十年中，肺癌诊断和治疗策略虽然取得了一定进步，但NSCLC患者的预后仍很差，5年总生存率仅为15%-20%^[6]^。这主要是由于缺乏早期诊断手段，有超过60%患者诊断时即为晚期或已发生转移性疾病^[7]^，因此没有获得外科切除治疗的机会。其主要原因为NSCLC早期阶段的诊断非常困难，且在以往临床诊断中我们很少应用到生物标记物。在NSCLC治疗中早期诊断是其治疗成功的一个关键因素，只有通过早期诊断后选择优化的治疗方案，才能增加其治愈成功的概率。因此，在临床上寻找NSCLC的早期筛查方法和手段尤为重要。近些年来，应用临床蛋白质组学策略发现NSCLC生物学标记物已成为临床NSCLC诊治的最新研究热点。我们不但可以通过它来筛查NSCLC高危人群，去发现那些可测定但临床上各项检测尚不能确诊的NSCLC，从而使NSCLC确诊的比率得到提高。而且，早期NSCLC预测性生物学标记物还可以帮助我们鉴别NSCLC的侵袭程度、提示我们哪些治疗会缩短患者生存率及帮助我们选择哪些患者会在辅助治疗中获益等作用。

尿液是可通过无创伤收集来应用于人类疾病诊断和预后研究的一种重要生物流体。因其具有实用性、易于收集和与疾病病理生理学相关等特性，已成为临床蛋白质组学一个最具有吸引力的材料来源。健康个体中，尿液蛋白质组的70%来自肾脏和尿路，30%来自肾小球滤过^[8]^。因此，尿液蛋白质组分析不仅能鉴定泌尿系统生物标志物，也能鉴定全身性疾病的生物标志物。最近许多研究工作表明，尿液蛋白质组学的研究还可为非泌尿生殖系统疾病，特别是恶性肿瘤提供大量生物学信息，并可使之应用于这些疾病的预防、诊断和治疗。通过对临床蛋白质组学研究的不断深入，尿液蛋白质组学可能会具有迅速在临床实用化的潜力。

本研究在NSCLC患者尿液蛋白质中发现了4种差异表达蛋白，分别为上调蛋白LRG1、CA1和下调蛋白VPS4B、YWHAZ。LRG1属于LRR蛋白质家族成员。在过去的研究表明，LRG1参与机体重要的生理和病理过程，如蛋白质相互作用、信号转导和细胞粘附。LRG1也可在粒细胞分化过程中得到表达。近年来研究发现肝癌^[9]^、肺癌^[10]^和胰腺腺癌^[11]^患者血清中LRG呈高表达。此外，Heo应用亲和层析色谱及LC-MS/MS对腺癌患者血清进行分离，鉴定出LRG1是一个潜在肺癌血清生物标志物的结论^[12]^。本实验结果表明LRG1在NSCLC患者尿液中呈高表达（敏感性83%，25/30；特异性90%，18/20），这说明NSCLC患者尿液中的LRG1蛋白可能是来自肺部肿瘤组织。同时也说明LRG1将有希望成为NSCLC相关的独立尿液生物学标志物。

碳酸酐酶（carbonic anhydrase, CA）同功酶在癌症发展中起到重要作用。其中一些同功酶可通过控制瘤体内pH值的平衡，呈现出调解恶性肿瘤细胞生物学行为的功能^[13]^。本研究发现在NSCLC患者的尿液中CA1蛋白质表达水平呈现明显上调（敏感性60%，18/30；特异性90%，18/20），而CA1过表达在结直肠癌^[14]^、口腔鳞状细胞癌^[15]^、子宫内膜癌^[16]^等恶性肿瘤研究中均有发现。因此，实验中这种变化可能反映了一定的NSCLC发生和发展过程，也为CA1蛋白可能成为NSCLC相关尿液生物学标记物，提供了理论依据。

VPS4B蛋白是腺苷三磷酸酶蛋白质家族的一员，主要存在于细胞浆中。VPS4B与多种细胞活性蛋白质相关，VPS4B通过与胞内体膜结合来调控激活膜蛋白内部和溶酶体降解^[17, 18]^。VPS4B是胞内体转运复合物所需（endosomal sortingcomplex required for transport, ESCRT）物质^[19, 20]^，其对多泡体（multivesicular body, MVBs）的形成和各种膜受体降解至关重要。膜受体包括表皮生长因子受体（epidermal growth factor receptor, EGFR）^[17, 21, 22]^和胰岛素受体^[23]^。表皮生长因子受体由多泡体转运，然后通过依赖VSP4B机制降解^[18]^。因此，VPS4B为ESCRT通路的重要调节器，并参与多泡体途径的组成。基于之前研究，VPS4B参与溶酶体降解途径，EGFR损失不仅导致内表皮生长因子受体退化延迟，同时也延长其激活持续时间^[24, 25]^，增加了肿瘤细胞增殖、生长、入侵和转移。已有VPS4B通过抑制EGFR激活途径抑制乳腺癌进展的报道^[26, 27]^。VSP4B不仅具有调节ESCRT通路中囊泡转运功能，还同样参与了病毒发生^[28, 29]^和胞质分裂的脱落^[30]^。有报道^[30]^称，VPS4B蛋白可以调节有丝分裂和细胞分裂的不同阶段。直到目前为止关于尿液VPS4B蛋白与NSCLC相关性研究几乎没有任何报道。但考虑到不受控制的细胞分裂是肿瘤形成的一个特征，因此很容易推测到VPS4B在NSCLC发生、发展中必将发挥一定的作用。本研究结果表明VPS4B在NSCLC患者尿液中呈现低表达（敏感73.3%，22/30；特异性90%，18/20），进一步证实这一假设的存在，也增加了尿液VPS4B蛋白作为NSCLC生物标记物的可能性。但关于其具体影响过程到目前为止尚不是完全清楚，有待于我们后续的实验研究来进一步解释。

YWHAZ蛋白质家族是通过绑定磷酸丝氨酸转载蛋白质进行基因调节信号转导。虽然，其在肿瘤发展中的潜在机制尚未完全清楚，但YWHAZ蛋白已在包括NSCLC在内的一些癌症中被确定为是一种假定的肿瘤蛋白。人体中确定出了YWHAZ蛋白家族中的7个亚型^[31]^，起初它们被认为是影响相关酪氨酸羟化酶活性的酶辅助因子^[32]^。但最近几项研究发现YWHAZ蛋白与原癌基因和癌基因相关，同时也参与了细胞转化和促进细胞有丝分裂的信号通路^[33, 34]^，对人体肿瘤的发生与发展产生广泛影响。YWHAZ蛋白一个明显特征是能够结合不同信号的多功能蛋白，包括激酶、磷酸酶和跨膜受体^[35]^。在这些蛋白相互作用的调控过程中YWHAZ发挥了重要作用，如引起细胞有丝分裂信号转导、细胞凋亡和细胞周期调控等^[36]^。到目前为止YWHAZ蛋白家族已经确定出超过100多个相关绑定蛋白^[37]^，同时在细胞、动物模型和患者样本中得到了越来越多的HWAZ与多种恶性肿瘤相关的证据，如神经母细胞瘤、星形细胞瘤及HeLa宫颈癌等。据赵^[38]^等报道，在NSCLC中YWHAZ与Hsp27结合成复合体，并对其功能产生重大影响。同时YWHAZ功能非常类似于Hsp27的作用功能。因此我们可以假设YWHAZ和Hsp27具有相同的信号通路来影响NSCLC的产生、发展过程。本研究发现NSCLC患者尿液中YWHAZ呈低表达（敏感性60.0%，18/30；特异性95%，19/20），进一步表明尿液YWHAZ蛋白的表达与NSCLC存在一定相关性。同时，我们更希望其成为一个独立预测NSCLC的新分子目标，从而服务于临床NSCLC的诊疗。

组成生物标记物的多肽或蛋白质在结构上存在明显可变性，且大部分经过机体排泄的多肽可能由于受到身体活动、饮食或药物治疗作用影响，其白天变化明显^[39, 40]^。所以单一生物标记物在临床中实用价值是有限的，即使采用最优的检测和分析方法，独立生物标志物应用于临床诊断中的敏感性依然很低。相比之下，单一生物标记变化不会导致标记物蛋白质组合的明显变化。因此，良好的多生物标记蛋白质组合具有更高的敏感性和特异性。这就进一步表明生物标志物蛋白质组合模式比独立生物标志物模式在临床诊疗评估中更有意义（如Theodorescu等^[41]^、Zimmerli等^[42]^），也将更具有良好的实用价值和前景。

实验研究中存在2处不足之处：①实验组NSCLC病例分期较晚；②对照组采用健康人群与肺部良性疾病。造成了独立生物标记物的特异性明显增高，故影响了其在临床意义上的作用。LRG1、CA1、VPS4B和YWHAZ作为NSCLC标记物在其他恶性肿瘤中的具体表达情况，还有待进一步的实验研究。
